# Ultrasonic Healing of Plastrons

**DOI:** 10.1002/advs.202403028

**Published:** 2024-07-01

**Authors:** Alex Drago‐González, Maxime Fauconnier, Bhuvaneshwari Karunakaran, William S. Y. Wong, Robin H. A. Ras, Heikki J. Nieminen

**Affiliations:** ^1^ Dept. of Neuroscience and Biomedical Engineering Aalto University Espoo Uusimaa 02150 Finland; ^2^ Dept. of Applied Physics Aalto University Espoo Uusimaa 02150 Finland

**Keywords:** acoustic radiation force, bubble, plastron, superhydrophobic surfaces, ultrasound

## Abstract

Superhydrophobic surfaces (SHS) exhibit a pronounced ability to resist wetting. When immersed in water, water does not penetrate between the microstructures of the SHS. Instead, a thin layer of trapped gas remains, i.e., plastron. This fractional wetting is also known as the Cassie–Baxter state (CB). Impairment of superhydrophobicity occurs when water penetrates the plastron and, when complete wetting is achieved, a Wenzel state (W) results. Subsequent recovery back to CB state is one of the main challenges in the field of SHS wetting. Current methods for plastron recovery require complex mechanical or chemical integration, are time‐consuming or lack spatial control. Here an on‐demand, contact‐less approach for performing facile transitions between these wetting states at micrometer length scales is proposed. This is achieved by the use of acoustic radiation force (ARF) produced by high‐intensity focused ultrasound (HIFU). Switching from CB to W state takes <100 µs, while the local recovery back to CB state takes <45 s. To the best of authors knowledge, this is the first demonstration of ARF‐induced manipulation of the plastron enabling facile two‐way controlled switching of wetting states.

## Graphical abstract

Acoustic radiation force is employed for manipulating the plastron of underwater micro‐pillared superhydrophobic surfaces. Focused ultrasound (US) facilitates controlled and localized reversible transitions between the Cassie–Baxter and the Wenzel states by collapsing the plastron or bulldozing millimeter‐scale bubbles. The methods can find use in micron scale systems in the fields of biomedicine and lab‐on‐a‐chip.

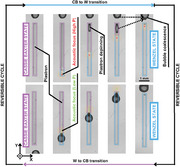

## 1 Introduction

Superhydrophobic surfaces (SHS) are extremely water‐repellent. Inspired by nature, e.g., the leaves of a lotus plant^[^ ^1^ ^]^ or the legs of water striders,^[^ ^2^ ^]^ synthetic SHS have been created using various micro‐ and nano‐fabrication techniques.^[^ ^3^ ^]^ SHS are characterized by a high Young's contact angle^[^ ^4^ ^]^ and a low contact angle hysteresis, i.e., the conditions for making surfaces dewetting.^[^ ^5^ ^]^

**Figure 1:**
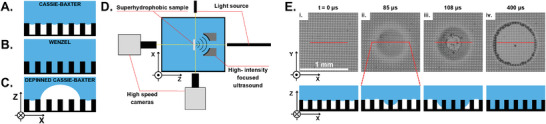
A) Schematic side view (XZ‐plane) of a pillar structured SHS underwater in CB wetting state; B) W wetting state; C). Depinned CB wetting state. D) Schematic representation of a side view (XZ‐plane) of the experimental setup. E) Experimental top‐view (XY‐plane) sequence (i–iv) and side‐view (XZ‐plane) schematic representation of a CB to W state transition induced by acoustic radiation force (ARF). (See Movie S1, Supporting Information).

Artificially designed SHS include micro‐ and nano‐structures,^[^ ^6^, ^7^, ^8^, ^9^, ^10^ ^]^ as a surface texture to enhance the coating properties, which will vary for different applications (e.g., anti‐icing,^[^ ^11^ ^]^ drag reduction,^[^ ^12^ ^]^ anti‐corrosion,^[^ ^13^ ^]^ shielding,^[^ ^14^ ^]^ coating,^[^ ^15^ ^]^ or self‐cleaning surfaces.^[^ ^16^ ^]^) When they are immersed in water, gas cavities are trapped between the solid domains of the SHS topography, forming a thin layer of gas. This composite layer is known as a plastron,^[^ ^17^ ^]^ physically representing the Cassie–Baxter state (CB)^[^ ^17^ ^]^ (**Figure** **1**A). However, because of the spontaneous hydrodynamic activity or external stimuli, superhydrophobicity can be compromised.^[^ ^18^ ^]^ If the gas–water interface moves toward the base of the SHS, the water penetrates the space between the microstructures and the surface becomes wet (Figure 1B), physically representing the Wenzel state (W).^[^ ^19^ ^]^ If the gas–water interface moves toward the liquid phase, the gas–water interface can detach, i.e., depinning,^[^ ^20^ ^]^ from the tips of microstructures such as micro‐pillars, forming a bubble that is tethered by its three‐phase contact line on the SHS (Figure 1C). This can be caused by defects on the microstructure^[^ ^21^ ^]^ or an increase of the intra‐plastronic pressure.^[^ ^22^, ^23^ ^]^ We understand this configuration as the depinned CB state.^[^ ^20^ ^]^

Underwater wetting transitions can be induced via passive methods (i.e., modifying the chemical composition^[^ ^24^, ^25^ ^]^ or the geometry of the surface^[^ ^26^, ^27^ ^]^) and active methods (i.e., boiling,^[^ ^28^ ^]^ hydrostatic pressure,^[^ ^22^, ^29^, ^30^ ^]^ electrochemical gas generation,^[^ ^31^ ^]^ or air injection through a porous substrate).^[^ ^32^ ^]^ Drawbacks of passive and boiling methods include long transition times and, in case of plastron restoration, only partial recovery is achieved. In addition to the previous methods, acoustics and vibration coupled with the solid SHS have been used to alter the wetting states.^[^ ^33^, ^34^, ^35^, ^36^ ^]^ However, literature studying the phenomena in submerged conditions is scarce.^[^ ^37^, ^38^ ^]^ These works have focused on describing the plastron collapse induced by the acoustic radiation force (ARF), i.e., a nonlinear phenomenon resulting from the interaction between an acoustic field and an object. For the investigated parameters (spatial‐peak, time‐averaged acoustic intensity *I*_SPTA_^[^ ^39^ ^]^ up to 5.0 kW cm^−2^ and driving frequency *f* = 1.1 MHz), their experimental results,^[^ ^37^ ^]^ validated by comparison to theoretical models of plastron collapse,^[^ ^40^, ^41^ ^]^ were limited to the CB to W transition and did not explore the capability of focused ultrasound (US) to induce plastron recovery.

ARF can occur when a wave meets an acoustic discontinuity.^[^ ^42^ ^]^ In this case, a radiation force,^[^ ^42^ ^]^ nonlinearly related to the acoustic pressure field, is generated. To characterize the driver for ARF exerted on the underwater SHS, we use the Langevin pressure $P_{\mathrm{Lan}}=\frac{I_{\mathrm{SPTA}}}{c_{0}}$, where *c*_0_ is the sound speed of the fluid carrying the wave.^[^ ^43^ ^]^

Currently, the standing challenge of rapid, localized, and reversible transition of the W to the CB remains. To solve this, focused nonlinear acoustics appears as a great candidate, given that it provides contact‐free and micron scale action. In that context, our study explores the spatiotemporal wetting dynamics of a plastron and the manipulation of SHS‐tethered bubbles to achieve facile reversible switchability of the W and CB wetting states, enabled by ARF. CB to W and W to CB wetting transitions are demonstrated via the acoustic manipulation of gas voids at the micrometer length scale.

## 2 Results and Discussion

### 2.1 Cassie–Baxter to Wenzel State Transition

In this study, the ARF produced by a focused US transducer (2.5 MHz, focal distance = 39 mm) is used to manipulate the gas–water interface of an underwater plastron. The experimental setup, shown in Figure 1D, allows to actuate the plastron's gas–water interface with a focused US field with a normal incidence.

As a preliminary result, we demonstrate that the CB to W state transition is achieved with an US pulse (See Table S1 and Section S1A in the Supporting Information document for details on the *I*_SPTA_ calculation, acoustic parameter details, and SHS dimensions) with lower *I*_SPTA_ than what has been reported in the literature (*I*_SPTA_ > 1.1 kW cm^−2^^[^ ^37^ ^]^) for pillar spacing 7.6 and 14.1 µm. Because increasing the pillar spacing lowers the impalement pressure,^[^ ^44^ ^]^ initiating the transition in our configuration (pillar spacing = 25 µm) is expected to happen at lower acoustic power and peak positive pressure (*PPP*). Figure 1E shows the details of this phenomenon via snapshots of the key events. Initially, at rest, we sonicate the gas plastron maintained by a SHS (See Table S1A Supporting Information). Originating from the epicenter of the actuation, the wetting then expands from there (Figure 1E‐iii). The final state, long after the US pulse stopped, reveals a collapsed plastron circle of ≈720 µm in diameter (Figure 1E‐iv) printed on the SHS, at the location of the acoustic focus. In this configuration, the plastron collapse typically occurs in a short time interval (<100 µs after the pulse impacted the plastron).

The spatiotemporal resolution to produce CB to W state transition through our ARF method is compared to the literature (See Section S1B and Figure S2, Supporting Information). We report higher spatiotemporal resolutions than previous methods.

We now use these principles to enable the plastron depinning, manipulation of SHS‐tethered bubbles and plastron recovery.

### 2.2 Plastron Depinning

**Figure 2:**
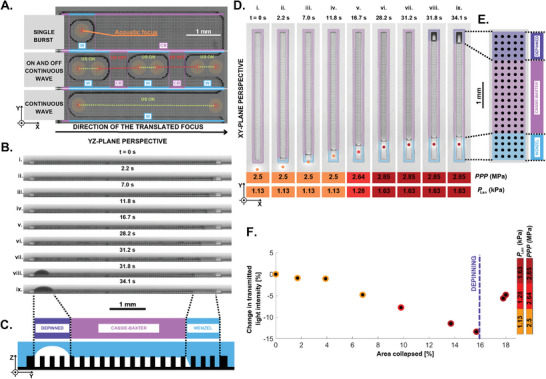
A) Experimental top view (XY‐plane) snapshots of an underwater SHS after a CB to W transition (collapse) was performed using the US actuation of a HIFU transducer. The transducer was not‐translated (top) or translated toward the right along a distance of 5 mm, while driven by an ON and OFF US sequence (middle) or a continuous wave (bottom). B) Experimental side view (YZ‐plane) of a rectangular SHS (See Table S1, Supporting Information) and C) its schematic representation. The progressive collapse advances from the right‐hand side forces the left‐hand side of the plastron to depin and form a bubble. D) Experimental top view (XY‐plane) of the depinning of plastron and the associated *PPP* and *P*_Lan_ (See Movie S3, Supporting Information), E) with a schematic representation of the phenomena. F) Graph of the relative percentage change from initial transmitted light intensity through the CB state due to the bulging of the gas–water interface during the depinning of the plastron as a function of the percentage of area collapsed.

One way to induce the plastron depinning is to increase the intra‐plastronic pressure, so that its gas–water interface bulges out and forms a bubble. This has been done before using an air injection system.^[^ ^45^ ^]^ Here, we propose an US‐based method not requiring any specific preparation of the SHS. To do so, the plastron is progressively collapsed using a continuous wave on a 5 mm × 5 mm SHS (See Table S1B, Supporting Information). In this case, the HIFU transducer is moved along the x‐axis using a translation stage and a collapsed trail is outlined with the translated location of the acoustic focus (**Figure** **2**A).

The same operation is performed in the case of a rectangular array of 225 µm × 5 mm and it results in what can be observed in Figure 2B,D. They respectively represent side‐ and top‐view sequences of experimental snapshots of the SHS being progressively collapsed from one end, together with side‐ and top‐view schematical representations of the final state (Figure 2C,E). In this process, we initially use a continuous wave (See Table S1C, Supporting Information) that eventually stops collapsing the plastron after 12 s of sonication. By increasing the acoustic pressure (See Table S1D, Supporting Information) we manage to further increase the W state area, while we observe that the CB state area turns optically darker (See Figure 2F). After 28 s of sonication, the ARF is not sufficient to keep collapsing the plastron again and an additional increase of the US power is required (See Table S1E Supporting Information). Finally, a SHS‐tethered bubble appears in the other end of the SHS (Figure 2B,D‐viii) and the optical appearance of the CB state area of the plastron returns to a brighter shade of gray (See Figure 2F).

This observed phenomenon can be explained due to the increase of the intra‐plastronic pressure when collapsing the plastron, which interferes with its stability^[^ ^45^ ^]^ and morphology.^[^ ^46^ ^]^ During the CB to W state transition, the plastron's volume is reduced and it requires a higher *P*_Lan_ to continue wetting the SHS. The increase in intra‐plastronic pressure also explains the darkening of the CB state area of the SHS, as the gas–water interface bulges out from the microstructure (See Section S1C and Figure S3, Supporting Information for an in‐depth explanation).

### 2.3 SHS‐Tethered Bubble Bulldozer

**Figure 3:**
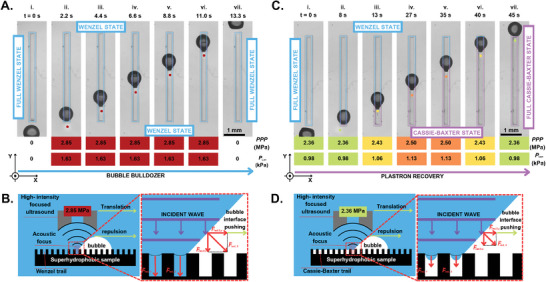
A–C). Demonstration of bubble translated by the means of ARF, along a superhydrophobic rectangular array. Initially in W state, a W trail or CB trail (See Movie S2, Supporting Information) is left upstream of the bubble, depending on whether a 2.85 MPa (A) or a 2.36–2.50 MPa (C) *PPP* is employed. The numerical values given at the bottom of each experimental snapshot informs on the estimated *PPP* and Langevin pressure at the acoustic focus. B–D). Side‐view sketches of the physics involved in each phenomenon.

With the same experimental configuration as elaborated in the previous section, we keep applying an US field of a continuous wave on the depinned plastron. By extending the collapsed area, the plastron continues to get depinned, eventually creating a SHS‐tethered bubble (Figure 2D‐viii,ix). Then, the ARF pushes the gas–water interface on one side of the bubble, facilitating a side‐ways translation. Simultaneously, it expels water out of the space in between the microstructures due to the increasing internal pressure arising from the ARF exerted on the other side of the bubble. This results in a pushing motion, i.e., bulldozing, on the bubble. This process is shown experimentally and schematically in **Figure** **3**A,B, where we present a top and side view of the translation of a bubble. In this visual demonstration, a continuous wave (See Table S1F, Supporting Information) is exceeding the collapse threshold, leaving a W state trail behind the bubble (Figure 3A).

### 2.4 Wenzel to Cassie–Baxter State Transition

One of the main challenges in the field of SHS wetting is the recovery of the CB state from the W state, i.e., dewetting. To do so, we reinstate gas into the wetted area of the SHS. This is achieved by using a continuous wave (See Table S1G, Supporting Information) working at a lower *P*_Lan_ than before, as we avoid the impalement of the SHS. The US field is used to transport a bubble along the rectangular SHS. By focusing the acoustic field on the trailing end of the bubble, we begin to push the bubble up the wetted channel, which is initially in W state (Figure 3C‐i–vii). An increased ARF pushes the gas–water interface out of the space in between the microstructures. By tuning the *P*_Lan_, we re‐establish the contact lines at the tips of the micro‐pillars, enabling us to regulate the plastron thickness (Figure 3D). Thus, after transporting the bubble along the SHS, the sample (that has an area of 1.125 mm^2^) is fully recovered to the CB state in 45 s, yielding a recovery rate of 2.5 × 10^−2^ mm^2^ s^−1^.

To our knowledge, this is the first demonstration of ARF‐induced recovery of plastron. Additionally, the spatiotemporal resolution to produce W to CB state transition through our ARF method is compared with other methods in the literature (See Section S1B and Figure S2, Supporting Information).

### 2.5 Reversible Switching Between Wetting States

**Figure 4:**
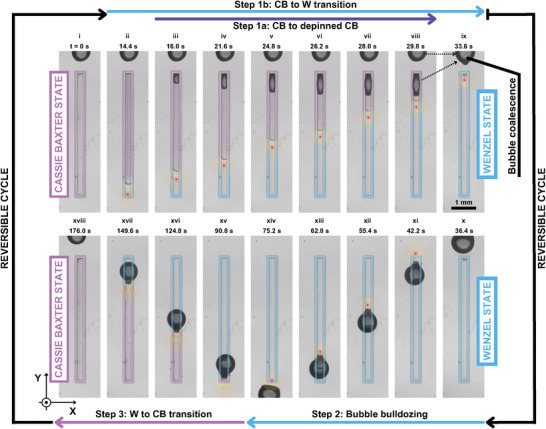
Top view (XY‐plane) of a cycle of experiments with the transition from an initial full CB state to partially depinned CB and W states, to a full W state, to final fully recovered CB state. The presented cyclic process has been made on a 225 µm‐wide, 5 mm‐long fluoro‐coated polydimethylsiloxane (PDMS) SHS with an array of 25 µm‐spaced, 20 µm‐wide, 50 µm‐tall cylindrical micro‐pillars. When the gas is removed from the rectangular SHS, the gas‐formed bubble merges with an external sessile bubble. This external bubble is needed to facilitate the W to CB state transition (See Movie S4, Supporting Information), which lasted less than 3 min.

Alternatively, we demonstrate 1) a W state from CB state transition, followed by a 2) recovery of the CB state from the W state. By combining the principles described in Figures 1E and 3A,C, a reversible transition cycle CB‐to‐W‐to‐CB was achieved (**Figure** **4**) in less than 3 min. The process was performed repeatedly on the same SHS. This implies that the acoustic field does not deteriorate the super water‐repellent properties of the SHS, allowing its reversible manipulation.

This process, performed on a rectangular SHS (225 µm × 5 mm), starts with the sonication of the bottom end of the SHS with a continuous wave during 16 s to progressively wet the sample, as described in Section 2.2 (Figure 4i–iii). Eventually, the plastron gets depinned on the top end of the SHS. We keep sonicating at the same acoustic power as before to accumulate all the gas from the plastron on a SHS‐tethered bubble, which is forming at the top end of the SHS (Figure 4iii–viii). This bubble is then pushed out of the array of micro‐pillars and is mixed with another bubble, which is resting a few micrometers away from the SHS (Figure 4ix). After the bubble coalescence, we push the resulting bubble until it is mounted on the top end of the rectangular SHS (Figure 4x–xi). Using the same acoustic power as before, we sonicate the trailing end of the bubble toward the bottom end of the SHS, leaving the surface in W state (Figure 4xi–xiv). Then, we mount again the bubble on the bottom end of the rectangular SHS. At the same time, we decrease the *P*_Lan_ to a value that is below the impalement pressure for the SHS, as we did in Section 2.4. Now, we push the bubble slowly toward the top end of the rectangular SHS (Figure 4xv–xviii). While pushing the bubble, small increases of the *P*_Lan_ are required in order to pierce the gas–water interface with the tips of the micro‐pillars. This adjustment guarantees that the SHS recovers the CB state with the gas–water interface repinned on the tips of the micro‐pillars, which is the same state that we found at the beginning of the cycle. The process finishes after 176 s of actuation and, from the last state, we can restart the process of turning the SHS from the initial CB state, to W state, to the recovered CB state.

It should be noted that, due to the ellipsoidal shape of the acoustic focus, there exists a minimum and a maximum distance between the SHS and the transducer (focal distance = 39 mm) where the switching between the CB and W states can be operated. Details are provided in Supporting Information (See Section S1D and Figure S4, Supporting Information).

### 2.6 Bubble Guidance Control

**Figure 5:**
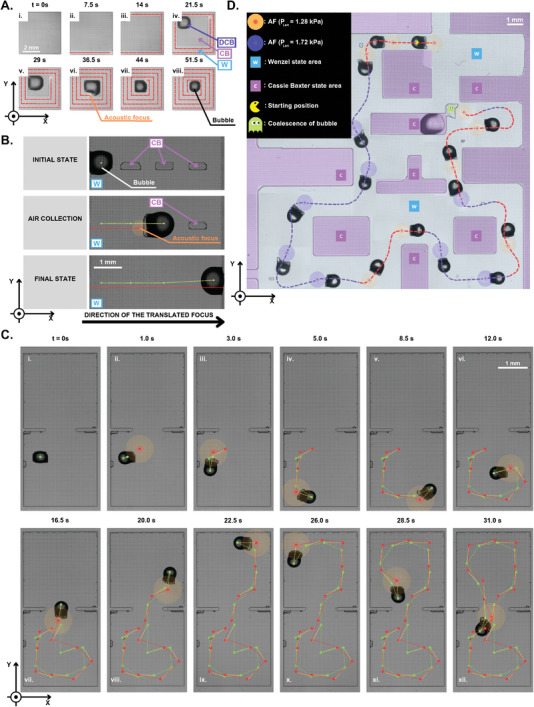
A) Top‐view (XY‐plane) sequence of the process of turning CB state gas into a SHS‐tethered bubble, where the US focus is translated along the dashed red line on SHS. B) Top‐view (XY‐plane) demonstration of an ultrasonically‐bulldozed SHS‐tethered bubble collecting plastron. C) Top‐view (XY‐plane) sequence (from i. to xii.) of a SHS‐tethered bubble translated in 2D from a one SHS‐chamber to another, connected by a 1 mm opening (See Movie S5, Supporting Information). D) Top view (XY‐plane) of a sequence of a bubble being ultrasonically bulldozed along a plastron‐made maze in a 2.5 cm by 2.5 cm squared sample, inspired by the arcade game of PAC‐MAN. The bubble follows a path in W state and the plastron‐made walls and obstacles, which cannot be touched by the bubble, are in CB state. The bubble is absorbed if it touches the plastron. The acoustic focus is shown for 0.19 (red) and for 0.25 kW cm^−2^ (purple).

Initially, we demonstrate how we turn a plastron into a SHS‐tethered bubble (**Figure** **5**A) using an US continuous wave (See Table S1H, Supporting Information), by collapsing the edges of a CB state area and pushing the gas toward the center of a SHS.

Then, we translate unidirectionally this bubble over CB state areas of the SHS with the same US continuous wave. The gas within the discontinuous plastron can be collected as shown in Figure 5B. This demonstrates how the bubble bulldozer could be steered to collect gas patches through their coalescence with the translated bubble.

To demonstrate the level of spatial control over the bulldozed bubble, we show two processes of transportation in two directions. This is achieved thanks to mounting the HIFU transducer on a two‐direction motion stage, allowing actuation along two axes. First, the bubble is translated with a continuous wave (See Table S1I, Supporting Information) at an average speed of 0.46 mm s^−1^ between two rectangular chambers separated by a wall and connected by a 1 mm‐wide opening (Figure 5C). Second, the bubble is guided through a plastron‐made maze inspired by the PAC‐MAN arcade game (Figure 5D). It features W state paths and CB state walls/obstacles created with a continuous wave (See Table S1J, Supporting Information) on a 2.5 cm × 2.5 cm SHS. In the latter example, a continuous wave (See Table S1K, Supporting Information) is used to bulldoze the bubble through the narrow W state path at an average speed of 0.31 mm s^−1^. Coalescence of the bubble with the plastron‐made wall is observed when it is forced to touch the obstacle in CB state (Figure 5D).

The results demonstrate how interactions of ARF and a SHS‐tethered bubble can be exploited to achieve spatiotemporal control over the bubble movement, maneuvered by adjusting the *P*_Lan_ and the position of the acoustic focus along the SHS.

## 3 Conclusion

In this work, we illustrate how an immersed plastron can be manipulated via acoustics (i.e., ultrasound) through a localized, contactless method. First, on‐demand reversible transitions between the Cassie–Baxter and Wenzel states were achieved, including the rarely demonstrated Wenzel‐to‐Cassie recovery. This was attained by the precise manipulation of millimetric bubbles on plastrons at micrometric length scales. Compared to the literature, our ARF approach is currently the only physical method allowing to switch locally between the SHS wetting states. Second, we show a delicate control over plastron height and curvatures (e.g., bulging or dimpling), enabled by spatiotemporally precise acoustic actuation. This technique offers new possibilities in numerous industrial and research settings (e.g., lab‐on‐chip systems for switching wetting states, gas voids transportation in submerged conditions, self‐cleaning of SHS, biomedical cell and particle manipulation).

## 4 Experimental Section

A sketch of the experimental setup is shown in Figure 1D. It consists of three systems: the superhydrophobic sample, the acoustic wave generation system (which both are immersed in an acrylic tank filled with pure water) and an external data acquisition system, which records the phenomena reported in this work.

### Superhydrophobic Surface Design

The superhydrophobic surface (SHS) made of polydimethylsiloxane (PDMS) comprised of cylindrical pillars (20 µm in diameter, 51 µm in height, 25 µm in wall‐to‐wall spacing) that were arranged in a square lattice, surrounded by a wall (50 µm‐thick and 50 µm‐tall). The two sizes of array employed in this work were 225 µm × 5 mm and 5 mm × 5 mm. The contact angle measurements were measured using a sessile drop method with a slightly modified protocol as in the literature.^[^ ^47^ ^]^ A conventional optical tensiometer (Attension Theta) installed with an automated water dispensing needle system was used. Advancing contact angle (ACA) measurement: first, a 2 µL drop was placed, and then, the drop volume was slowly increased to 12 µL drop at a rate of 0.05 µL s^−1^. Receding contact angle (RCA) measurement: first, a 15 µL drop was placed on the surface, and then, the drop volume was decreased at a rate of 0.05 µL s^−1^. When the drop volume decreased below 2.5 µL, the stick‐and‐slip motion became prominent. The RCA reported here was averaged across the smallest contact angle for each oscillation. The measured advancing and receding contact angles of the PDMS pillars coated with PFOTS [Trichloro(1*H*,1*H*,2*H*,2*H*per‐fluorooctyl) silane, 97%, Sigma–Aldrich] were 165° ± 3°and 115° ± 3°, respectively. For the plain PDMS (no pillars) coated with PFOTS, the advancing and receding contact angles were 121 ± 3° and 103 ± 3°, respectively.

### Microfabrication

The samples were fabricated in PDMS using a micropatterned silicon wafer as a master. The wafer was initially spin‐coated with a commercially available photopolymer (SU‐8 50, Micro resist technology) and baked. MLA 150 (Heidelberg) was the tool used for exposing the coated wafer using a laser (λ = 375 nm, exposure dosage of 200 mJ cm^−2^ and defocus of 0). The laser beam was programmed to move according to a 2D design pattern fed into the tool. The design pattern, which was made in KLayout 0.27.2 to generate a GDSII file, consisted of a series of circles (diameter 20 µm, corresponding to the pillars width) and a series of lines (50 µm‐wide, corresponding to the walls that surround the 2D arrays). After exposure, the wafer was baked and developed. It was baked once again (hard bake, at 170°C for 20 min on a hot plate). The wafer was then coated with a thin layer of fluoropolymer using reactive ion etching (Oxford Instrument, Plasma RF generator) with CHF_3_ (at 99.5 cm^3^ min^−1^ for 15 min), at a chamber (pressure of 250 mTorr and a DC bias of 80 V). The micropatterned structures obtained at this stage were a negative of the intended pillar structures.

The monomer and the curing agent of PDMS (Sylgard 184, Dow Corning) were mixed in a ratio of 10:1 and degassed. The mixture was poured on the patterned silicon wafer, degassed until no more air bubbles were visible, and then baked (80 °C for 3 h) in a hot‐air oven. The cured PDMS was peeled off gently from the mold. The fluoropolymer on the silicon master enabled easy peeling of molded PDMS. The PDMS sample was subjected to oxygen plasma (10 min in Diener electronic plasma chamber). The sample was quickly transferred to a desiccator (100µL of PFOTS) inside. The desiccator was left in vacuum (for 10 min) for chemical vapor deposition of PFOTS. After that time, the PFOTS container was removed, and the PDMS sample was left in vacuum condition in the same desiccator (for 30 min) before being used for experiments. The pillar heights were obtained by measuring the depth of the SU‐8 structures on the patterned silicon wafer using a stylus profilometer (DektakXT, Bruker).

### Superhydrophobic Dipping Protocol

The SHS was dipped in pure water (MilliQ, 18.2 *M*Ωcm at 25 °C, 1 ppb total organic carbon at initial temperature ranging from 28.1 to 28.7 °C) at speed (lower than 10 mm s^−1^) to ensure a reproducible thickness of the plastron.^[^ ^48^ ^]^ The SHS was held by a three‐axis optometric station, allowing micrometric displacements of the sample in any direction.

### Ultrasound Actuation

The acoustic actuation was generated by a high‐intensity focused US (HIFU) transducer, which was dipped in the pure water and focused on the SHS with the pillars facing the transducer (while perpendicularly oriented against the gravity vector in the Y direction). The HIFU transducer (Sonic Concepts Inc., Model:H‐147; f = 2.5 MHz; focus width at –6 dB: 0.51 mm; focus length at ‐6 dB: 3.28 mm, Washington, USA) generated continuous and pulsed waves, depending on the intended phenomena. The length and duration of the wave used in this study are specified in Table S1, Supporting Information, for each reported phenomena. The HIFU transducer could move in the XY‐plane thanks to a manual two‐axis translation stage (Standa Ltd., Model: 7T175‐100, Lithuania) and had a hole in the middle, which enabled a light source to illuminate through it to observe the phenomena from a bottom view perspective. The US pulse and the continuous wave (input peak‐to‐peak voltage in the range of 1.4–3 V) was generated by a signal generator (B&K Precision, Model:4053b, California, USA) and amplified 50 times by a high voltage amplifier (Falco Systems B.V., Model:WMA‐300, Netherlands).

### High‐Speed Imaging

The surface dynamics were monitored by a high‐speed camera (Vision Research, Model:Phantom v1612, New Jersey, USA) with varying frame rates (from 200 to 77 000 fps) depending on the phenomena and it was controlled through the software Phantom Camera Control (Phantom, v3.4). The camera was equipped with a 5x magnification objective (Canon Inc., Model:MP‐E 65 mm, Japan) and the optical focus was matched in the same position as the acoustic focus, enabling clear observation of the pillars and phenomena at the gas–water interface. As shown in Figure 1D, the camera had two positions to observe the phenomena with two light sources in the contrary side of the acrylic water tank. The first one showed the SHS from a bottom‐perspective in the XY‐plane and allowed an easy recognition of the position of the acoustic focus. The second showed the SHS from a side‐view perspective in the YZ‐plane and allowed observation of the bulging of the plastron (including the formation of the SHS‐tethered bubble).

## Conflict of Interest

The authors declare no conflict of interest.

## Supporting information

Supporting Information

Supplemental Movie 1

Supplemental Movie 2

Supplemental Movie 3

Supplemental Movie 4

Supplemental Movie 5

## Data Availability

Curated data is available from the corresponding author upon request.

## References

[1] W. Barthlott , C. Neinhuis , Planta 1997, 202, 1.

[2] X. Gao , L. Jiang , Nature 2004, 432, 36. 15525973 10.1038/432036a

[3] J. Jeevahan , M. Chandrasekaran , G. Britto Joseph , R. Durairaj , G. Mageshwaran , J. Coat. Technol. Res. 2018, 15, 231.

[4] T. Young , Philos. trans. R. Soc. Lond. 1805, 95, 65.

[5] G. Carbone , L. Mangialardi , Eur. Phys. J. E. 2005, 16, 67. 15688142 10.1140/epje/e2005-00008-y

[6] K. K. S. Lau , J. Bico , K. B. K. Teo , M. Chhowalla , G. A. J. Amaratunga , W. I. Milne , G. H. McKinley , K. K. Gleason , Nano Lett. 2003, 3, 1701.

[7] L. Feng , S. Li , Y. Li , H. Li , L. Zhang , J. Zhai , Y. Song , B. Liu , L. Jiang , D. Zhu , J. Adv. Mater. 2002, 14, 1857.

[8] T. Onda , S. Shibuichi , N. Satoh , K. Tsujii , Langmuir 1996, 12, 2125.

[9] S. Shibuichi , T. Onda , N. Satoh , K. Tsujii , J. Phys. Chem. 1996, 100, 19512.

[10] D. Wang , Q. Sun , M. J. Hokkanen , C. Zhang , F.‐Y. Lin , Q. Liu , S.‐P. Zhu , T. Zhou , Q. Chang , B. He , Q. Zhou , L. Chen , Z. Wang , R. H. A. Ras , X. Deng , Nature 2020, 582, 55. 32494077 10.1038/s41586-020-2331-8

[11] L. B. Boinovich , A. M. Emelyanenko , V. K. Ivanov , A. S. Pashinin , ACS Appl. Mater. Interfaces. 2013, 5, 2549. 23470194 10.1021/am3031272

[12] M. A. Samaha , H. V. Tafreshi , M. G. el Hak , CR MECANIQUE 2012, 340, 18.

[13] T. Ishizaki , Y. Masuda , M. Sakamoto , Langmuir 2011, 27, 4780. 21417352 10.1021/la2002783

[14] D. Ashok , M. Taheri , P. Garg , D. Webb , P. Parajuli , Y. Wang , B. Funnell , B. Taylor , D. C. Tscharke , T. Tsuzuki , N. K. Verma , A. Tricoli , D. R. Nisbet , Adv. Sci. 2022, 9, 2201415. 10.1002/advs.202201415 PMC9376840 35657076

[15] K. Zheng , J. Zhang , E. Keaney , H. Dodiuk , S. Kenig , C. Barry , J. Mead , J. Coat. Technol. Res. 2021, 18, 685.

[16] Y.‐B. Park , H. Im , M. Im , Y.‐K. Choi , J. Mater. Chem. 2011, 21, 633.

[17] A. B. D. Cassie , S. Baxter , Trans. Faraday Soc. 1944, 40.

[18] M. S. Bobji , S. V. Kumar , A. Asthana , R. N. Govardhan , Langmuir 2009, 25, 12120. 19821621 10.1021/la902679c

[19] R. N. Wenzel , Ind. Eng. Chem. 1936, 28, 988.

[20] X. W., C. CH , Phys. Rev. Lett. 2012, 109, 024504. 23030167 10.1103/PhysRevLett.109.024504

[21] C. Liu , H. Zhan , J. Yu , R. Liu , Q. Zhang , Y. Liu , X. Li , Surf. Coat. Technol. 2019, 361, 342.

[22] P. Forsberg , F. Nikolajeff , M. Karlsson , Soft Matter 2011, 7, 104.

[23] A. Sarkar , A.‐M. Kietzig , Soft Matter 2015, 11, 1998. 25627327 10.1039/c4sm02787f

[24] W. S. Y. Wong , D. Vollmer , Adv. Funct. Mater. 2018, 32, 2107831.

[25] D. Panchanathan , A. Rajappan , K. K. Varanasi , G. H. McKinley , ACS Appl. Mater. Interfaces. 2018, 10, 33684. 30184437 10.1021/acsami.8b12471

[26] T. Verho , J. T. Korhonen , L. Sainiemi , V. Jokinen , C. Bower , K. Franze , S. Franssila , P. Andrew , O. T. Ikkala , R. H. A. Ras , Proc. Natl. Acad. Sci. U.S.A. 2012, 109, 10210. 22689952 10.1073/pnas.1204328109 PMC3387048

[27] B. P. Lloyd , P. N. Bartlett , R. J. K. Wood , ACS Omega 2021, 6, 3483. 33644523 10.1021/acsomega.0c03466 PMC7906494

[28] F. Barghi , M. Entezari , S. Chini , A. Amirfazli , Int. J. Heat Mass Transf. 2020, 156, 119705.

[29] A. Cannon , in Int. Conf. Solid‐State Sensors, Actuators and Microsystems (Transducers), IEEE, New York 2009, pp. 362.

[30] W.‐K. Lee , W.‐B. Jung , S. R. Nagel , T. W. Odom , Nano Lett. 2016, 16, 3774. 27144774 10.1021/acs.nanolett.6b01169

[31] R. Freeman , in Int. Conf. on Solid‐State Sensors, Actuators and Microsystems (Transducers), IEEE, Anchorage 2015, pp. 1818.

[32] J. Breveleri , S. Mohammadshahi , T. Dunigan , H. Ling , Colloids Surf. A: Physicochem. Eng. Asp. 2023, 676, 132319.

[33] E. Bormashenko , R. Pogreb , G. Whyman , Y. Bormashenko , M. Erlich , Appl. Phys. Lett. 2007, 90, 201917.

[34] J. B. Boreyko , C.‐H. Chen , Phys. Rev. Lett. 2009, 103, 174502. 19905763 10.1103/PhysRevLett.103.174502

[35] W. Lei , Z.‐H. Jia , J.‐C. He , T.‐M. Cai , G. Wang , Appl. Phys. Lett. 2014, 104, 181601.

[36] Z.‐h. Jia , W. Lei , H. Yang , G. Wang , Adv. Mater. Sci. Eng. 2016, 2016, 1.

[37] A. Bussonnière , Q. Liu , P. Tsai , J. Fluid Mech. 2023, 956, A32.

[38] B.‐E. Pinchasik , H. Wang , H. Möhwald , H. Asanuma , Adv. Mater. Interfaces 2016, 3, 1600722.

[39] W. R. Hedrick , D. Hykes , J.Diagn. Med. Sonogr. 1991, 7, 188.

[40] S. Moulinet , D. Bartolo , Eur. Phys. J. E. 2007, 24, 251. 18060595 10.1140/epje/i2007-10235-y

[41] M. Reyssat , J. M. Yeomans , D. Quéré , EPL 2007, 81, 26006.

[42] A. Doinikov , Acoustic radiation forces: Classical theory and recent advances, Transworld Research Network, Kerala, India 2003.

[43] F. A. Duck , A. Baker , H. Starritt , Ultrasound in Medicine, CRC Press, Florida 1998.

[44] H.‐J. Butt , C. Semprebon , P. Papadopoulos , V. Doris , M. Brinkmann , M. Ciccotti , Soft Matter 2012, 9, 418.

[45] W. Cho , S. Heo , S. J. Lee , Phys. Fluids 2022, 34, 122115.

[46] P. Lv , Y. Xiang , Y. Xue , H. Lin , H. Duan , Phys. Fluids 2017, 29, 032001.

[47] T. Huhtamäki , X. Tian , J. T. Korhonen , R. H. Ras , Nat. Protoc. 2018, 13, 1521. 29988109 10.1038/s41596-018-0003-z

[48] H. de Maleprade , C. Clanet , D. Quéré , Phys. Rev. Lett. 2016, 117, 094501. 27610858 10.1103/PhysRevLett.117.094501

