# Supplementary material for: Ultrasonic Healing of Plastrons
Source: Adv Sci (Weinh). 2024 Jul 1;11(33):2403028. doi: 10.1002/advs.202403028 (PMC11434134; doi:10.1002/advs.202403028)
Supplement: Supplementary file 1 — Supporting Information [file ADVS-11-2403028-s005.pdf]

ADVANCED SCIENCE
Open Access

Supporting Information

for *Adv. Sci.*, DOI 10.1002/advs.202403028

Ultrasonic Healing of Plastrons

Alex Drago-González, Maxime Fauconnier, Bhuvaneshwari Karunakaran, William S. Y. Wong, Robin H. A. Ras and Heikki J. Nieminen**

Supporting Information for the document entitled:
Ultrasonic healing of plastrons

This document contains the supporting information for the manuscript titled "*Ultrasonic healing of plastrons*", submitted in *Advanced Science* on the 22nd of March of 2024.

---

1. SUPPORTING SECTIONS

A. MATLAB code to compute acoustic parameters

To compute the spatial-peak, time-averaged acoustic intensity (I_{SPTA}), we measured the pressure field at the focal point with a hydrophone, when the power amplifier had a driving peak-to-peak voltage ranging from 1 to 4 V. First, we use MATLAB to convert the instantaneous pressure into instantaneous intensity. Then, we define an interval for the time-averaged integration of the intensity. In this case, we used three cycles from the measured signal after the recorded signal had plateaued. From the results of I_{SPTA}, the Langevin pressure (P_{Lan}) has been calculated as follows:

$$P_{\mathrm{Lan}} = \frac{I_{\mathrm{SPTA}}}{c_0} \quad , \tag{S1}$$

where c_0 is the speed of sound in water [1]. Finally, we have used a polynomial fit of form $y = Ax^2$ to interpolate I_{SPTA} and P_{Lan} values at any selected peak-to-peak driving voltage within the range 1-4V. To do this, we obtained a least-squares fit and we have presented the graph of the given function together with the measured values (See Figure S1).

```
% Read files
datav = cell(1, 4);
datav{1} = dlmread('1Vpp.txt');
datav{2} = dlmread('2Vpp.txt');
datav{3} = dlmread('3Vpp.txt');
datav{4} = dlmread('4Vpp.txt');

% Hydrophone characteristics:
M = 70e-9; % Sensitivity [1e-9V/Pa = 1mV/MPa] at 2.5MHz

% Initialize variables
t = cell(1, 4);
p = cell(1, 4);
I = cell(1, 4);

% Constants
rho = 998; % Density (water) [kg/m^3]
c = 1485;  % Sound speed (water) [m/s]

% Processing data
for i = 1:4
    t{i} = datav{i}(:, 1); % Time [s]
    p{i} = datav{i}(:, 2) / M; % Pressure [Pa] over time
    I{i} = (p{i}.^2) / (rho * c); % Intensity [W/m^2] over time
end

% Interval of integration for pulse duration
frequency = 2.5e6; % Frequency [Hz]
cycles = 3; % Limited the study around on 3 cycles after the signal plateaued
time_interval = cycles / frequency; % Time interval of integration [s]

% Calculation of spatial-peak, temporal-averaged intensity and Langevin Pressure:
PLancalc = [];
intcalc = [];

for i = 1:4
    % Find the maximum intensity and its index
    [max_intensity, idx] = max(I{i});
    max_time = t{i}(idx);

    % Delimitation of the integration
    idxini = idx - 2; % Beginning of the first cycle
    idxfin = idx + 22; % End of the last cycle

    % Calculate spatial-peak, time-averaged intensity
    p_squared = p{i}.^2;
```

```
    I_spta = trapz(t{i}(idxini:idxfin), p_squared(idxini:idxfin)) / (rho * c * time_interval);
    intcalc = [intcalc, I_spta];  % Add I_spta to the intcalc list

    % Calculate Langevin Pressure
    PLan = I_spta / (c); % PLan in Pa
    PLancalc = [PLancalc, PLan];  % Add PLan to the PLancalc list
end

% Display the results lists
disp('Spatial-peak pulse-averaged intensities: ');
disp(intcalc);
disp('Langevin Pressures: ');
disp(PLancalc);

%% Plotting results
voltage = [1 2 3 4]; % driving voltage (Vpp)
xq = 0:0.05:voltage(end);

% Quadratic adaptation:
X = voltage.^2;

% Perform linear regression to fit y = a*x^2
A = X';

% Squares solution to the linear system A*a = y
a = A \ PLancalc';  % '\' operator solves the linear equation
b = A \ intcalc';

% Display the coefficients
disp('Coefficient of x^2 of Langevin pressure:');
disp(a);
disp('Coefficient of x^2 of acoustic intensity:');
disp(b);

% Mathematical approximation of Langevin Pressure and Acoustic intensity
y2 = a*xq.^2; % PLan = I/c [Pa]
y3 = b*xq.^2; % I = c*PLan [W/m^2]

% Plot of Langevin pressure
figure
plot(voltage, PLancalc, 's','MarkerSize',10, 'MarkerEdgeColor','red', 'MarkerFaceColor',[1 .6 .6])
xlabel('driving voltage [V]')
ylabel('Langevin pressure [Pa]')
hold on
plot(xq, y2)
xlim([0, 4.5]);
ylim([0, 8.5]);


% Plot of spatial-peak, temporal-averaged acoustic intensity
figure
plot(voltage, intcalc, 's','MarkerSize',10, 'MarkerEdgeColor','blue', 'MarkerFaceColor',[0 0 1])
xlabel('driving voltage [V]')
ylabel('Acoustic intensity [W/m^2]')
hold on
plot(xq, y3, 'b-')
xlim([0, 4.5]);
```

Listing 1. Plotting and calculating acoustic intensity and Langevin pressure.

In Table S1 we show how we have varied those two parameters (*i.e.*, I_{SPTA} and P_{Lan}) for each experiment, together with the dimensions of the superhydrophobic surface (SHS).

B. Comparison to other methods

The numerical comparison between each method, including our ultrasound (US) approach, is presented in Table S2 and Figure S2, including all the known studies for underwater wetting transitions. In our case, the lateral resolution is defined by the width of the acoustic focus (AF), *i.e.* beam diameter (BD) as follows [2]:

$$BD \simeq 1.02\frac{\lambda R}{D} = 1.02\frac{c_0 R}{Df} \tag{S2}$$

where λ is the wavelength, R is the radius of curvature of the transducer, D is the aperture (*i.e.*, diameter of the transducer opening) and c_0 is the speed of sound in the medium carrying the wave. In our case, the BD is 0.51 mm. The lateral resolution of our method could be enhanced if the BD could be smaller, which could in principle be achieved by increasing the transducer frequency, decreasing the radius of curvature of the transducer or increasing the transducer aperture size.

Also, note that the boiling and electrochemical methods are not present in the left image of Figure S2. That is because the Cassie-Baxter (CB) to Wenzel (W) transition is not claimed in both studies.

C. Light refraction due to plastron bulging

One of the main observations during the process of depinning the plastron was the reduction of light intensity of the CB state area. In Figure S3 we show our understanding of this effect. Initially, the air-water interface is slightly curved towards the bottom of the SHS [3]. In this configuration, the light is refracted [4] (*i.e.*, less photons arrive to the sensor of the camera), making the high-speed camera to record a light shade of gray (Figure S3, i).

When collapsing the sample, the volume of the plastron is reduced. Therefore, the intra-plastronic pressure rises. This causes the air-water interface to bulge towards the liquid, making the light to refract again [4] (Figure S3, ii).

If we keep collapsing the plastron laterally, the bulging increases. Consequently, the light intensity is reducing due to an increased refraction of the light [4] (Figure S3, iii).

This process ends when the bulging of the air-water interface becomes too pronounced, making the interface to depin from the tips of the micropillars. When this happens, a SHS-tethered bubble appears. Additionally, the bulging relaxes and the shade of gray recorded by our camera turns lighter (Figure S3, iv). Then, the bulging slowly returns to the initial state.

D. Maximum switching distance

There is a maximum distance from the transducer in which we can switch between the CB and W state.

In our study, we use a transducer that has a focal distance of 39 mm. In order to guarantee the same acoustic effect in all the experiments, the distance between the SHS and the transducer has been fixed at 39 mm. At that distance, we are able to heal the plastron using lower acoustic power compared to the collapse. The cycle between these two phenomenon results into the switching of the wetting state of the plastron. Because the CB to W transition requires a higher acoustic pressure to happen than the W to CB, the switching process is limited in the z-axis (direction in which the distance between the transducer and the SHS is defined) by the collapse. Therefore, the maximum switching distance from the transducer will be restricted by the furthest distance in which we can collapse the plastron at a specific acoustic power.

The US focus, spatially defined as the region of half maximum power, has an ellipsoid shape of length 3 mm. This means that the operating distance of US to switch between the CB and the W states corresponds, approximately,to 39 ± 1.5 mm. This is experimentally demonstrated in Figure S4. In this figure, we show that a plastron collapse is achieved if the plastron is located within the distance range of 39 ± 1 mm from the transducer aperture, for the employed driving parameters (400 µs single pulse at $I_{\mathrm{SPTA}} = 0.67$ kW cm^{-2}). Elsewhere, the plastron remains intact or can be slightly distorted at the edge of the AF (37.5 and 40.5 mm from the transducer).

2. SUPPORTING FIGURES

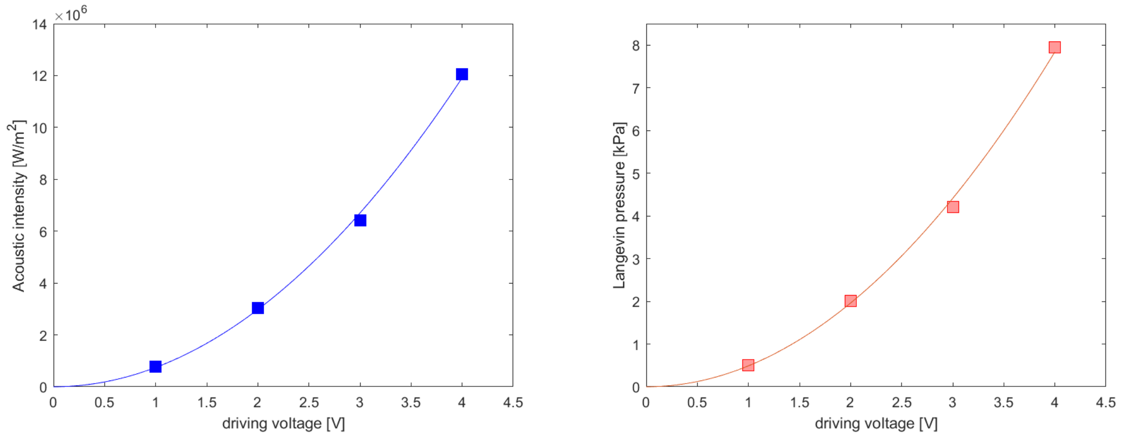

Fig. S1. I_{SPTA} (Left) and P_{Lan} (Right) as a function of the driving peak-to-peak voltage given by the wave generator. The driving voltage is amplified 50×, when arriving to the transducer.

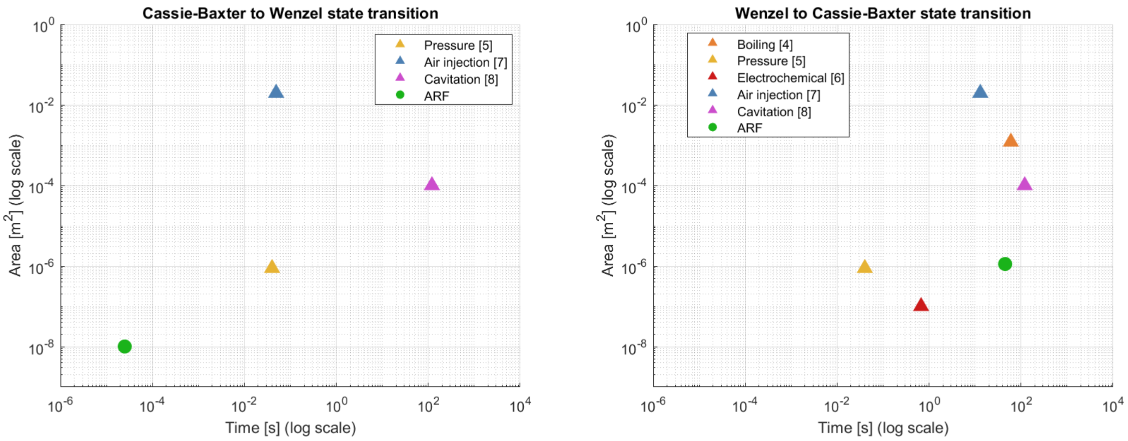

Fig. S2. Comparison of the space and time resolution of each active method compared to ours (green), when doing a transition from Cassie-Baxter to Wenzel state (Left) and vice versa (Right). The space resolution is given in m^2 units and the time resolution is given in s, with both axis in logarithmic scale. In circular shape, are presented the methods that allow a localized actuation on wetting. In triangle shape, are presented those methods that the space resolution is driven by the spatial limitation of the SHS used. ARF refers to acoustic radiation force used in the present study.

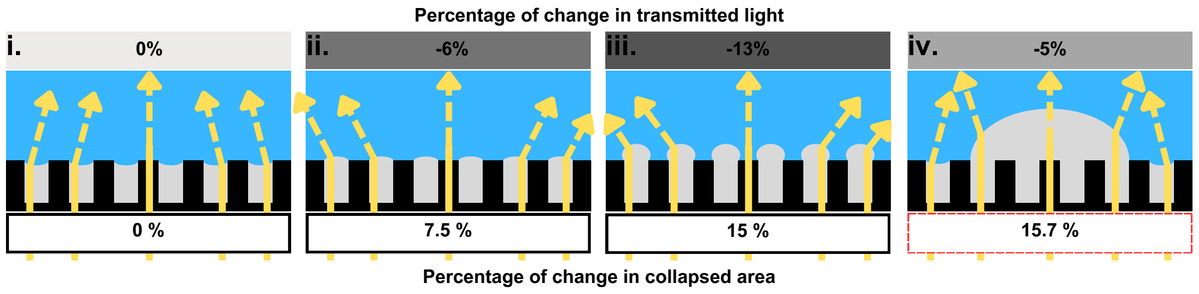

Fig. S3. Evolution of the light refraction due to the bulging of the air-water interface during the process of depinning plastron.

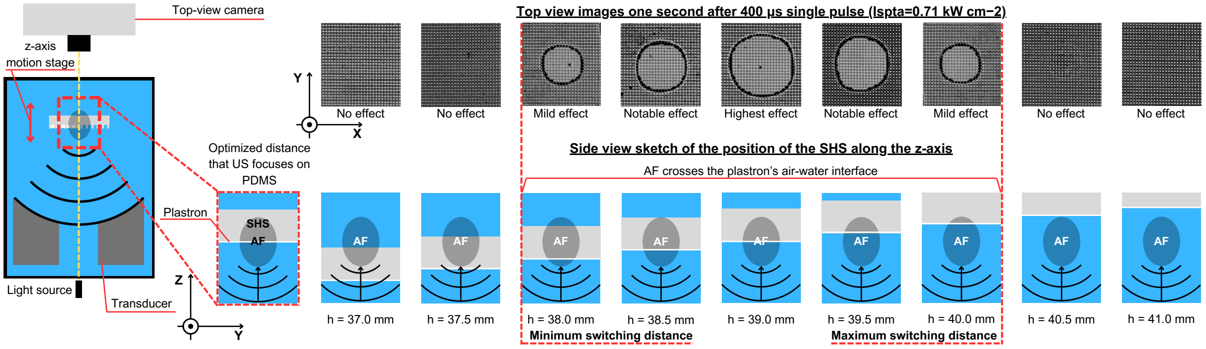

Fig. S4. Evaluation of the maximum switching distance from the transducer. On the left, we show the experimental setup used, where a z-axis motion stage has been incorporated to the SHS holder. On the top row, we show a top view of the resulting wetting state of the SHS after a 400 μs single pulse at a I_{SPTA} = 0.67 kW cm^{-2}. We see that the optimized effect is achieved at the focal distance (39 mm), but the effect is noticeable between a minimum (38 mm) and maximum (40 mm) distance. On the bottom row, we represent a schematic side view of how we place the SHS at different distances of the transducer, making the air-water interface of the plastron to be crossing (or not) the AF.

3. SUPPORTING TABLES

Table S1. US 2.5 MHz wave characteristics for each one of the experiments reported in this study. The calculation of I_{SPTA} is described in Section S1,A, P_{Lan} is measured from Equation S1.

Experiment	Sample size	Wave mode	*PPP* [MPa]	I_{SPTA} [kW cm^{-2}]	P_{Lan} [kPa]
A. Collapse	(5×5) mm^2	400 μs single pulse	4.6	0.67	4.52
B. Depinning 1	(5×5) mm^2	continuous	2.50	0.17	1.13
C. Depinning 2	(5×0.225) mm^2	continuous	2.50	0.17	1.13
D. Depinning 3	(5×0.225) mm^2	continuous	2.64	0.19	1.28
E. Depinning 4	(5×0.225) mm^2	continuous	2.85	0.24	1.63
F. Bulldozing	(5×0.225) mm^2	continuous	2.85	0.24	1.63
G. Recovery	(5×0.225) mm^2	continuous	2.36 - 2.50	0.14 - 0.17	0.98 - 1.13
H. Guidance 1	(5×5) mm^2	continuous	2.92	0.25	1.72
I. Guidance 2	(2.5×5) mm^2	continuous	2.92	0.25	1.72
J. Guidance 3	(25×25) mm^2	continuous	2.92	0.25	1.72
K. Guidance 4	(25×25) mm^2	continuous	2.6 - 2.92	0.19 - 0.25	1.28 - 1.72

Table S2. Underwater wetting transitions comparison. *indicates porous media.

**Trans,**	**Res.**	[5]	[6]	[7]	[8]	[9]	[10]	ARF
	Height	> 50 μm*	9.5 μm	150 μm	10-50 μm*	1 μm	5 μm	50 μm
**CB-W**	Time	-	> 40 ms	-	49 ms	120 s	< 100 μs	< 100 μs
	Spatial	-	0.88 mm^2	-	19.63 cm^2	1 cm^2	33.18 cm^2	0.01 mm^2
**W-CB**	Time	> 1 min	> 40 ms	670 μs	13 s	120 s	-	< 45 s
	Spatial	> 1200 mm^2	0.88 mm^2	0.1 mm^2	19.63 cm^2	1 cm^2	-	1.13 mm^2

4. SUPPORTING MOVIES

Movie M1. Top view of a CB to W state transition.

Movie M2. Top view of a W to CB state transition.

Movie M3. Top view the process of depinning the gas-water interface from the tips of the micropillars.

Movie M4. Top view demonstration of the reversible cycle from CB to W to CB.

Movie M5. Top view demonstration of the guidance of a SHS-tethered bubble.

REFERENCES

1. N. Bilaniuk and G. S. Wong, "Speed of sound in pure water as a function of temperature," J Acoust Soc Am **93**, 1609 (1993).
2. T. Kundu, Ultrasonic and Electromagnetic NDE for Structure and Material Characterization: Engineering and Biomedical Applications (CRC Press, 2012).
3. X. W. and C. CH., "From sticky to slippery droplets: dynamics of contact line depinning on superhydrophobic surfaces." Phys. Rev. Lett. **109**, 024504 (2012).
4. F. Bryant, "Snell's law of refraction," Phys. Bull. **9**, 317 (1958).
5. F. Barghi, M. Entezari, S. Chini, and A. Amirfazli, "Effect of initial wetting state on plastron recovery through heating," Int. J. Heat Mass Transf. **156**, 119705 (2020).
6. P. Forsberg, F. Nikolajeff, and M. Karlsson, "Cassie–wenzel and wenzel–cassie transitions on immersed superhydrophobic surfaces under hydrostatic pressure," Soft Matter **7**, 104 (2011).
7. R. Freeman, "Visualization of self-limiting electrochemical gas generation to recover underwater superhydrophobicity," in Int. Conf. on Solid-State Sensors, Actuators and Microsystems (Transducers), (Anchorage, 2015), p. 1818.
8. J. Breveleri, S. Mohammadshahi, T. Dunigan, and H. Ling, "Plastron restoration for underwater superhydrophobic surface by porous material and gas injection," Colloids Surf. A: Physicochem. Eng. Asp. **676**, 132319 (2023).
9. B.-E. Pinchasik, H. Wang, H. Möhwald, and H. Asanuma, "Fully reversible transition between cassie and wenzel states via acoustic waves," Adv. Mater. Interfaces **3**, 1600722 (2016).
10. A. Bussonnière, Q. Liu, and P. Tsai, "Acoustic responses of underwater superhydrophobic surfaces subjected to an intense pulse," J. Fluid Mech. **956** (2023).
